# Genes, gut microbiome and lungs: untangling the web of idiopathic pulmonary fibrosis

**DOI:** 10.1183/23120541.00204-2026

**Published:** 2026-08-24

**Authors:** Tamara Hernandez-Beeftink, Richard J. Allen, Olivia C. Leavy, Leah Cuthbertson, Philip L. Molyneaux, Louise V. Wain

**Affiliations:** 1 Division of Public Health and Epidemiology, School of Medical Sciences, University of Leicester, Leicester, UK; 2 University Hospitals of Leicester NHS Trust, Leicester, UK; 3 Division of Microbiology and Infection, School of Biological Sciences, University of Leicester, Leicester, UK; 4 National Heart and Lung Institute, Imperial College London, London, UK

## Abstract

Idiopathic pulmonary fibrosis (IPF) is a poorly understood chronic lung disease characterised by progressive fibrosis, with poor survival and limited treatment options. IPF is thought to result from an aberrant response to lung injury, culminating in an exaggerated healing response with excessive deposition of extracellular matrix in the interstitium [1]. Genetic studies have emphasised high heritability and common polygenic aetiology in the development of IPF. There are more than 30 replicated common genetic signals associated with IPF [1], implicating genes involved in host defence, cell proliferation, and signalling, among others.

## Shareable abstract

**This work highlights the use of genetics to explore potential causal links between the gut microbiome and IPF, underscoring the importance of integrating gut and lung microbiome data with host genetics to better understand IPF mechanisms** https://bit.ly/4bdzf4V

*To the Editor:*

Idiopathic pulmonary fibrosis (IPF) is a poorly understood chronic lung disease characterised by progressive fibrosis, with poor survival and limited treatment options. IPF is thought to result from an aberrant response to lung injury, culminating in an exaggerated healing response with excessive deposition of extracellular matrix in the interstitium [1]. Genetic studies have emphasised high heritability and common polygenic aetiology in the development of IPF. There are more than 30 replicated common genetic signals associated with IPF [1], implicating genes involved in host defence, cell proliferation, and signalling, among others.

The lung harbours a diverse microbial community even in the absence of infection, and changes in its composition have been associated with disease progression in IPF [2]. Candidate gene studies of acute exacerbations-related death in IPF identified a specific genetic variant in *TLR3*, which plays a critical role in pathogen recognition and innate immunity activation, deregulates the lung microbiome in IPF and reduces lung fibroblast responses [3]. This suggests that it may have a role in mortality associated with acute exacerbations induced by viruses and bacteria. In addition, the strongest genetic risk factor for IPF (the *MUC5B* promoter variant, rs35705950) was independently associated with lung bacterial burden in IPF patients [4]. However, studies investigating the effects of host genetics on the airway or lung microbiome in health or disease are still needed, as previous studies have been limited by sample size.

The gut microbiome has emerged as a promising area of investigation in respiratory diseases, due to its critical role in regulating immune responses and maintaining systemic homeostasis [5]. Dysbiosis in gut microbiome composition can influence inflammation and immune modulation, potentially affecting disease progression. People with IPF have been shown to have altered gut microbiota profiles compared to healthy controls, characterised by decreased diversity and changes in the abundance of specific bacterial taxa [6]. Moreover, animal studies support the role of the lung–gut axis in IPF pathogenesis, and suggest that altering the gut microbiota through antibiotics, probiotics, or faecal microbiota transplantation can moderate the severity of lung fibrosis [7].

A study of the effect of host genetics on gut microbiome composition in the general population (N=18 340) identified 31 genetic loci influencing the microbiome at genome-wide significance (*p*<5×10^−8^) [8]. Interestingly, one of the signals was located near the *FUT2* gene, which has been shown to affect exacerbation frequency and airway microbiota in patients with non-cystic fibrosis bronchiectasis [9]. This signal has also been associated with lung function and chronic sputum production in the general population [10]. Mendelian randomisation studies have shown evidence of potential causal relationships between specific gut bacterial taxa and IPF susceptibility and lung function, therefore supporting the importance of the gut microbiome in IPF and suggesting that interventions targeting microbiome-related pathways might have therapeutic benefit [11].

Our aim was to identify shared genetic signals involved in IPF pathogenesis and gut microbiome composition that could highlight specific causal links. Given the limited availability of gut microbiome data from IPF patients, we took genetic associations with gut microbiome composition in the general population and assessed overlap with genetic associations with IPF susceptibility, progression (measured using lung function decline) and transplant-free survival times after IPF diagnosis.

Of 31 genetic regions previously associated with the gut microbiome regulation [8], two signals were nominally (*p*<0.05) associated with IPF susceptibility (5159 IPF cases and 27 459 controls) [1], and a further four were nominally associated with IPF disease progression (including lung capacity (forced vital capacity) and gas transfer (diffusing capacity of the lung for carbon monoxide); N=1048 individuals with IPF). The most significant association was an intronic signal in the *NTRK2* gene, previously implicated in fibroblast activation in pulmonary fibrosis [12], which was associated with slower lung function decline in IPF (n=1 048, β=31.2 mL·year^−1^ (95% CI 7.5–54.9), p=6.62×10^−3^), and a decrease in the bacterial genus *Oxalobacter* in the general population (table 1, upper half). These results are exploratory, and should be interpreted with caution given the nominal significance, and the limited sample sizes for progression analyses.

**TABLE 1:** Association results

Rsid-effect allele	Nearest gene^#^	Gut microbiome			Idiopathic pulmonary fibrosis		
		Trait	Z-score^¶^	p-value	Trait	OR or β^#^ (95% CI)	p-value
**Gut microbiome signals nominally associated with IPF**							
rs9864379-T	*LSM3*	family.unknownfamily.id.1000001214 genus.unknowngenus.id.1000001215 order.Gastranaerophilales.id.1591	−5.46 −5.46 −5.46	4.66×10^−8^ 4.66×10^−8^ 4.66×10^−8^	Risk (1)	0.92 (0.85,0.99))	0.033
rs9864379-T	*LSM3*	family.unknownfamily.id.1000001214 genus.unknowngenus.id.1000001215 order.Gastranaerophilales.id.1591	−5.46 −5.46 −5.46	4.66×10^−8^ 4.66×10^−8^ 4.66×10^−8^	Longitudinal FVC^§^ [PMID: 35985358]	−38.4 (−74, −2.8)^+^	0.032
rs736744-T	*NTRK2*	genus.Oxalobacter.id.2978	−5.57	2.57×10^−8^	Longitudinal FVC^§^ [PMID: 35985358]	31.2 (7.5,54.9)^+^	6.62×10^−3^
rs12781711-C	*MIR6072*	genus.RuminococcaceaeUCG013.id.11370	−5.57	2.55×10^−8^	Longitudinal *D*_LCO_^ƒ^ [PMID: 35985358] Longitudinal FVC^§^ [PMID: 35985358]	−25.2 (−48.9,−1.5)^+^ −0.05 (−0.10,0.00)^+^	0.030 0.035
rs437867-A	*OR1F1*	genus.LachnospiraceaeUCG.010.id.11330.binary	5.52	3.34×10^−8^	Risk (1)	0.95 (0.90–1.00)	0.037
rs67476743-T	*CNN2*	genus.Tyzzerella3.id.11335	5.90	3.74×10^−9^	Longitudinal FVC^§^ [PMID: 35985358]	25.2 (−0.9–51.3)^+^	0.043
**IPF risk signals associated with gut microbiome GWAS after applying Bonferroni correction**							
rs9426886-T	*TRIM46*	genus.Eubacteriumventriosumgroup.id.11341	−3.27	1.07×10^−3^	Risk (1)	1.16 (1.11,1.22)	1.53×10^−9^
rs2736100-A	*TERT*	genus.LachnospiraceaeUCG004.id.11324 genus.LachnospiraceaeUCG001.id.11321	3.46 3.25	5.35×10^−4^ 1.15×10^−3^	Risk (1)	1.37 (1.30,1.44)	7.86×10^−36^
rs1214761-A	ZNF318 (6p21.2)	genus.Erysipelatoclostridium.id.11381	−3.37	7.42×10^−4^	Risk (1)	0.85 (0.81,0.90)	1.32×10^−9^
rs35705950-T	*MUC5B*	genus.Eubacteriumxylanophilumgroup.id.14375	3.46	5.46×10^−4^	Risk (1)	5.22 (4.89,5.58)	<5.00×10^−121^
rs3742238-T	*ATP11A*	family.Peptococcaceae.id.2024	−4.10	4.06×10^−5^	Risk (1)	0.78 (0.74,0.83)	3.22×10^−14^
rs2304645-G	*IVD*	genus.Butyricicoccus.id.2055	3.89	9.88×10^−5^	Risk (1)	0.79 (0.75,0.83)	1.10×10^−21^
rs112087793-T	*STMN3*	genus.Ruminococcustorquesgroup.id.14377	−3.68	2.32×10^−4^	Risk (1)	0.78 (0.71,0.86)	2.40×10^−7^

OR: odds ratio (for idiopathic pulmonary fibrosis (IPF) risk); 95% CI: 95% confidence interval; FVC: forced vital capacity; *D*_LCO_: diffusing capacity of the lung for carbon monoxide; GWAS: genome-wide association study. ^#^: Based on ANNOVAR v07.06.20; ^¶^: Overall Z-score (beta/se) from Kurilshikov et al. [13]; ^+^: β (beta): effect size (for IPF progression); ^§^: ml/year for longitudinal FVC; ^ƒ^: mmol/min/kPa/year for longitudinal *D*_LCO_.

24 of 35 previously reported association signals for IPF risk were at least nominally significant for association with at least one gut bacterial taxon. Seven signals were significant after accounting for 35 tests (p<1.4×10^−3^), although none would have passed a more stringent threshold also accounting for 211 taxa; 7385 tests, p<6.77×10^−6^ (table 1, lower half).

The most significant shared signal (p=4.06×10^−5^) was a single nucleotide polymorphism (SNP) at the *ATP11A* gene associated with decreased IPF risk (rs3742238-T) and with decreased relative abundance of the *Peptococcaceae* family. The second most significant signal (p=9.88×10^−5^) was a SNP allele at the Isovaleryl-CoA Dehydrogenase (*IVD*) gene associated with decreased IPF risk (rs2304645-G) and with increased relative abundance of *Butyricicoccus* genus. To identify potential shared causal variants between gut microbiome variation and IPF, we performed colocalisation analysis using the coloc R package (https://github.com/chr1swallace/coloc). Both signals showed a high posterior probability (PP) of shared a causal variant contributing to IPF risk and *Peptococcaceae* (PP=0.945) and *Butyricicoccus* (PP=0.918) relative abundance. These taxa have not previously been linked to IPF.

Some treatments have been shown to modulate the gut microbiome in pulmonary fibrosis. For example, the administration of a traditional medicine used for cough and sputum production (*Amygdalus mongolica* oil) in animal models increased intestinal diversity and the abundance of several bacterial taxa, including *Peptococcaceae*, which was associated with reduced oxidative and inflammatory damage and attenuation of fibrosis severity [5]*.* Although this appears to contradict our finding of a genetic link between increased fibrosis risk and *Peptococcaceae* abundance, this may reflect context-dependent effects, whereby shifts in the microbiome associated with disease susceptibility differ from those observed during therapeutic modulation. This association warrants further investigation using higher taxonomic resolution to clarify the specific role of *Peptococcaceae* in fibrosis.

The *ATP11A* gene encodes an integral membrane ATPase involved in the innate immune response, with reduced protein levels linked to increased inflammation [13]. The IVD enzyme plays a crucial role in breaking down the amino acid leucine within the mitochondria, an essential process for generating energy and preventing the accumulation of toxic by-products. Previous studies in inflammatory bowel disease have suggested that maintaining *Butyricicoccus* levels may reduce disease risk or slow progression [14]. This raises the possibility that a similar intervention might have benefit for individuals with IPF or those at high risk of disease [15]. Further investigation is needed to clarify the relationships between the genetic association signals and microbiome composition to establish whether they reflect causal effects or pleiotropy.

In conclusion, our study leveraged existing large-scale, population-based data in a hypothesis- generating framework to identify shared genetic associations of gut microbiome composition and IPF risk. Key limitations include reliance on publicly available summary-level data, and limited taxonomic resolution. Detection of host genetic–microbiome associations requires large sample sizes to achieve adequate statistical power, highlighting the current lack of IPF-specific microbiome cohorts with paired genetic information that are large enough to study what occurs after disease onset. Therefore, our findings should not be interpreted as evidence of causality, but rather as indicating potential shared genetic mechanisms that warrant further investigation. We propose that systematic collection of gut microbiome and genetic data in well-characterised IPF cohorts is essential to validate these associations, enable pathway-level analyses, and determine whether interventions targeting the gut microbiome could meaningfully influence IPF risk, progression, or prevention.

## Data Availability

The idiopathic pulmonary fibrosis genome-wide association study (GWAS) data are available from https://github.com/genomicsITER/PFgenetics. The gut microbiome GWAS data in the general population are available from www.mibiogen.org.
